# Complex challenges of estimating the age and vitality of muscle wounds: a study with matrix metalloproteinases and their inhibitors on animal and human tissue samples

**DOI:** 10.1007/s00414-021-02563-6

**Published:** 2021-05-26

**Authors:** A. Niedecker, R. Huhn, St. Ritz-Timme, F. Mayer

**Affiliations:** 1 Institute for Legal Medicine at the University Hospital Essen, 45147 Essen, Germany; 2 Departement of Anesthesiology, the University Hospital Düsseldorf, 40225 Düsseldorf, Germany; 3 Institute for Legal Medicine at the University Hospital Düsseldorf, 40225 Düsseldorf, Germany

**Keywords:** Wound age, Wound vitality, Immunohistochemistry, Myocardium, Skeletal muscle

## Abstract

The estimation of wound age and wound vitality is a recurring task in forensic routine work and has been subject of forensic research for a long time. By now, an unrestrictedly reliable marker or set of markers has not been found. In a study on myocardial infarctions, matrix metalloproteinases (MMP) 2 and 9 as well as tissue inhibitor of matrix metalloproteinases 1 (TIMP-1) were detected immunohistochemically in mechanically wounded myocardium (ECG electrodes, vessel ligations). Against this background, the potency of MMP-9, MMP-2, and TIMP-1 as markers for the estimation of wound age and wound vitality was tested in a broad approach with human tissue samples drawn during autopsies and with an animal model, the isolated perfused Langendorff heart. The study comprised samples of injured human skeletal muscle, injured human myocardium, rats’ hearts with vital wounds, and rats’ hearts with postmortem-inflicted wounds that were all stained immunohistochemically. The results showed great scattering, leading to the conclusion that MMP-2, MMP-9, and TIMP-1 are not suitable for wound age estimation. Merely the results for TIMP-1 suggested that this marker might be able to differentiate between vital and postmortem-inflicted wounds. With a view to the promising results of the preceding study, the results underline the necessity to test possible markers of wound age/wound vitality on a large and diverse sample set.

## Introduction

Reconstructing events of physical violence by evaluating the point of time when wounds have been inflicted is a recurring task in forensic routine work. Not surprisingly, wound age estimation has been a central field of forensic research for a long time.

Once injured by force, the body tissues respond with numerous molecular and cellular reactions in order to fix the damage: Wound healing is activated. The chronological course of wound healing can be divided in different phases, which are characterized by various processes and mediators [1]. Estimating the wound age and/or the vitality of wounds is based on identifying the phases of wound healing by detecting these processes/mediators and putting them into a temporal context. Besides evaluating the macroscopic appearance of an injury, wound age estimation also comprises an assessment of microscopic and molecular alterations.

A recent review by Li et al. [2] stated that progress towards a more precise estimation of vitality and the age of wounds has been made during the last years. However, the authors also point out that an unrestrictedly reliable marker or set of markers has not been identified yet. Research problems are mainly seen in the availability and quantity of human tissue samples with sufficient information on wound age and wound vitality, as well as the results’ reproducibility, the examiner’s experience and methodological limitations. Casse et al. [3] arrive at a similar conclusion. According to their study, research on wound age estimation and wound vitality demands the consideration of various factors to ensure not only high specificity and sensitivity, but also the reproducibility of the results. They underline the necessity of control groups and the differentiation between antemortem and postmortem wounds with a simultaneously adequate number of samples.

This necessity derives from the phenomenon of the so-called biological death, meaning the death of each single cell a certain time after the death of the organism, the “individual death”. In 2010, Alaeddini et al. [4] stated that the interval of survival varies in different body tissues due to their own survival mechanisms. In this time span, some physiologic processes might still go on and bias the estimation of wound age. They might even suggest vitality of an injury that has actually been inflicted postmortem. A review by Dunjić et al. [5] also suggests that the activity of cells after the individual death depends on the type of tissue. Consequently, transferring research findings from a specific type of wound to other body tissues is nearly impossible [4].

A recent study of our own research group on myocardial infarctions came up with positive immunohistochemical staining for matrix metalloproteases (MMP) 2 and 9 and their inhibitor TIMP-1 (tissue inhibitor of matrix metalloproteases) not only in ischemic areas, but also adjacent to wounds inflicted mechanically by electrodes or vessel ligations in rats’ hearts [6]. In contrast, myocardial samples without an injury, ischemia, or other determinable harms (increased workload due to pulmonary embolism, cardiac resuscitation) represented no (increased) staining. MMPs are zinc-requiring proteolytic enzymes that are synthetized as zymogens, meaning in a proactive form, and are activated after an injury [7]. In healthy tissue, MMPs are expressed continuously on a low level. Their expression increases when tissues are being remodeled [8], in physiologic as well as pathologic processes [7]. They play a central role especially in the early phase of wound healing by degrading extracellular matrix (EM) [9].

In humans, more than twenty different, class-divided MMPs have been identified [10], one important sub-group are the gelatinases (MMP-2 and MMP-9). Due to repeats homologous to fibronectin type II in their catalytic domains, they specifically degrade different types of collagens in the EM [11]. High levels of MMP-2 and MMP-9 were detected in wounds after surgery and in chronic wounds [12, 13]. Furthermore, studies on skin samples and studies on ovarian carcinoma cells have shown that MMP-9 influences wound healing by activating TGF-beta via proteolysis and inducing the expression of vascular endothelial growth factor (VEGF) [14, 15]. A study on MMP-2 knockout mice was able to show that MMP-2 plays a key role in angiogenesis and tumor progression [16]. The activation of MMPs is complex and strictly regulated on multiple stages [17]. The primary control instance are TIMPs, a family of four enzymes (TIMP-1 to TIMP-4). They inhibit MMPs and thus inhibit the degradation of EM. TIMP-1 has a high affinity to MMP-9 and its inactive form, progelatinase B [18]. The interaction of MMPs and TIMPs seems to be important for wound healing [19]. In a review, Conlon et al. [8] stated that MMPs not only act as proteolytic enzymes and inductors of the expression of signal molecules; moreover, they induce the activation of other MMPs. The different ways of MMP activation seem to intertwine. Previous studies also indicate that the ratio between MMPs and TIMPs is of great importance for wound healing. Ladwig et al. [19] showed that the ratio of MMP-9 and TIMP-1 could be used as an indicator of wound healing in wound fluid of pressure ulcers. A dysregulation of MMPs and TIMPs seems to be one of the reasons why wound healing is defective in chronic wounds [20]. In a study on dermal wounds, Gillard et al. [9] discovered elevated expression of MMP-9, MMP-2, and TIMP-1 especially in early phases of wound healing, concluding that MMP-9 might be important for angiogenesis, whereas MMP-2 might play a role in tissue transformation.

In addition to our own research results [6], all these findings suggest that a closer look on the applicability of MMPs and TIMPs in the context of wound age estimation and wound vitality could be worthwhile. Though promising, our findings in the preceding study [6] only gave a hint that MMPs and TIMPS can be detected immunohistochemically in the early phase of wound healing of myocardial tissue—other important questions, however, remained unanswered:

- Can MMP-2, MMP-9, and TIMP-1 be detected immunohistochemically not only in injured myocardium, but also in skeletal muscle?
- If so, does their occurrence depend on the “age” of the examined wounds?
- Are there differences between the two types of muscle tissue?
- Can the markers help to differentiate between vital and postmortem-inflicted wounds?

We aimed on addressing these questions with a broad and complex approach. The immunohistochemical detectability of MMP-2, MMP-9, and TIMP-1 was examined in two types of injured muscle tissue, myocardium and skeletal muscle. Moreover, we worked with postmortem drawn, human tissue samples and with an animal model, the isolated perfused Langendorff heart. This model allowed us to generate myocardial wounds with a defined “age” as well as postmortem-inflicted wounds.

## Materials and methods

Animal experiments were performed in accordance with the German legislation on protection of animals and the Guide for the Care and Use of Laboratory Animals published by the US National Institutes of Health (NIH Publication No. 85–23, revised 1996). The protocol for the Langendorff system was approved by the local Animal Ethics Committee (project no. O 27/11). The examination of human myocardium and skeletal muscle samples drawn during autopsies was approved by the ethical committee of the Medical Faculty of the Heinrich-Heine-University Düsseldorf (project no. 5833).

### Human study samples

A total of 208 tissue samples of muscle wounds, skeletal muscle (140 samples) and myocardium (68 samples), were selected from 141 autopsies at the Department of Legal Medicine at the University Hospital Düsseldorf, Germany, in the period from 2006 to 2017. We included different types of violence (strangulation, blunt force, sharp force, polytrauma, myocardial injuries due to surgery, and myocardial injuries due to infarction). The age of the tissue donors ranged between 16 months and 94 years and both sexes were included.

In a first step, wound age of each sample was estimated roughly according to the available data (assumed time period between infliction of wound and death of the individual). The estimate was refined by additionally considering hematoxylin&eosin (HE) staining results and classifying the findings according to Cummings et al. [21]:

- A: very short survival time, few min max.—no signs of inflammation, no neutrophilic infiltration
- B: few min up to 4 h—single perivascular neutrophils
- C: 4 h up to 8 h—enhanced neutrophilic infiltration
- D: 8 h up to 12 h—infiltration of neutrophils, macrophages, and fibroblasts

### Isolated perfused Langendorff heart

We used white male Wistar rats aged 2–3 months. The weight ranged between 250 and 350 g. The preparation of the rats’ hearts was performed as described before [6]: The rats were kept on a 12:12 light/dark schedule (lights on at 0600 h) with food and water ad libitum. The animals were anesthetized by intraperitoneal injection of Pentobarbital (90 mg kg^−1^) and Heparin (0.2 ml). The depth of sedation was verified by the absence of reactions to pain. In this state, the rats were decapitated, an immediate thoracotomy was conducted and hearts were excised and mounted onto the Langendorff system. The hearts were perfused with modified Krebs–Henseleit-Buffer:

- 118 mM sodium chloride (VWR Chemicals Prolabo)
- 4.7 mM potassium chloride (Fluka)
- 1.2 mM magnesium sulfate hepta-hydrate (Sigma-Aldrich)
- 1.2 mM potassium dihydrogen phosphate (Merck)
- 25 mM sodium hydrogen carbonate (Roth)
- 0.5 mM ethylenediaminetetraacetic acid (Roth)
- 11 mM D-glucose (VWR Life Science and Roth)
- 1 mM L-lactic acid sodium salt (Serva)
- 2.25 mM calcium chloride (Merck)

Heart function was monitored by observing heart rate, intraventricular pressure, and electrocardiogram (ECG). For data digitalization, we used an analog to digital converter (PowerLab/8SP, ADInstruments Pty Ltd., Castle hill, Australia) with a sampling rate of 500 Hz. Data documentation was carried out frequently by using Chart for Windows v5.0 (AD-Instruments).

### Rats’ hearts with vital wounds

**Fig. 1:**
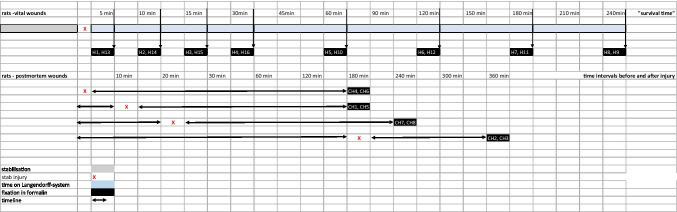
Study protocols for rats’ hearts with vital wounds (H1-H16) and with postmortem-inflicted wounds (CH1-CH8). Hearts with vital wounds were mounted onto the Langendorff system, injured by stabbing the left chamber after a stabilisation period of 20 min and fixed in formalin after different “survival times”. Hearts with postmortem-inflicted injuries were injured by stabbing the left chamber. Time intervals before and after stabbing varied

After a stabilization period of about 20 min on the Langendorff system, 16 hearts were injured by stabbing the wall of the left chamber with a scalpel. After defined time intervals of 5, 10, 15, 30, 60, 120, 180, and 240 min, the hearts were removed from the Langendorff system and directly immersed in 4% formalin. An overview of the study protocol is given in Fig. 1.

### Rats’ hearts with postmortem-inflicted wounds

Eight hearts were excised after decapitation without being attached to the Langendorff system. After a defined time interval, they were injured by stabbing the wall of the left chamber with a scalpel: Two hearts each were stitched immediately, 10 min and 180 min after they had stopped beating; after another 180 min, the six hearts were fixed in 4% formalin. Furthermore, two hearts were injured 20 min after they had stopped beating and were fixed in 4% formalin after another 240 min (see also Fig. 1).

### Immunohistochemical analysis

Identical staining methods were used for both human study samples and rat study samples and were performed as described before by Mayer et al. [6]:

Tissue sections were deparaffinized, washed in distilled water three times for 5 min and washed in TBS buffer with 0.5% Tween 20 two times for 5 min.

- MMP-2: Slides were boiled in citrate buffer pH 6.0 for 10–15 min, cooled, and then washed in distilled water two times for 5 min. Primary antibody against MMP-2 (Medac, rabbit, E 18,012) was used in a concentration of 1:200, and the slides were incubated over night at + 4 C. Slides were washed in TBS buffer with 0.5% Tween 20 two times for 5 min. Endogenous peroxidase was blocked with 0.03% H2O2 for 20–25 min.
- MMP-9: Slides were treated with proteinase K (Dako, S3020) for 7 min and washed two times in TBS buffer with 0.5% Tween 20 for 5 min. Primary antibody against MMP-9 (Biorbyt orb, rabbit, 13,583) was used in a concentration of 1:300, and the slides were incubates over night at + 4 C. Slides were washed in TBS buffer with 0.5% Tween 20 two times for 5 min. Endogenous peroxidase was blocked with 0.03% H2O2 for 10–15 min.
- TIMP-1: Primary antibody against TIMP-1 (Biorbyt orb, rabbit, 195,994) was used in a concentration of 1:300, and the slides were incubated over night at + 4 C. Slides were then washed in TBS buffer with 0.5% Tween 20 two times for 5 min. Endogenous peroxidase was blocked with 0.03% H2O2 for 10–15 min.

After blocking of endogenous peroxidase, all slides were washed in distilled water two times for 5 min and then in TBS buffer with 0.5% Tween 20 two times for 5 min. Afterwards, all slides were incubated with a peroxidase-marked polymer (Medac, Histofine1 Simple Stain MAX PO against rabbit, 414,142) for 30 min. Slides were stained with AEC (3-Amino-9-Ethylcarbazole, Cohesion Biosciences) and counterstained with Mayers hematoxylin (Merck, HX87717149).

### Evaluation system for immunohistochemical analysis

To standardize the results of the immunohistochemical analysis, we used the following evaluation system as published before [6]:

MMP-9 and TIMP-1:

- 0: No visible staining
- I: Positive staining of single cells
- II: Positive staining of cell groups
- III: Positive staining of large tissue areas

MMP-2:

- 0: No visible staining
- I: Positive staining of EM in the perivascular regions
- II: Positive staining of EM in larger areas
- III: Positive staining not only of EM but also of intracellular

## Results

### General observations

Whereas positive staining for MMP-9 and TIMP-1 was found strictly intracellular, positive staining reactions for MMP-2 were also found in the EM. These findings correlate with the results of Mayer et al. [6].

Slides from human samples that had been stored in formalin for a longer time showed less distinct staining results compared to those samples collected more recently (2015–2017). In order to exclude a relevant influence of storage time on our results, we evaluated the slides of the “older” cases separately and compared them to the “younger” ones without finding any differences. We also checked, if the causative type of violence has an impact on the occurrence of the markers, again, no differences were found. Therefore, the results in this publication comprise all collected samples without separating them into the different types of violence for a clearer depiction.

### Human skeletal muscle

**Fig. 2:**
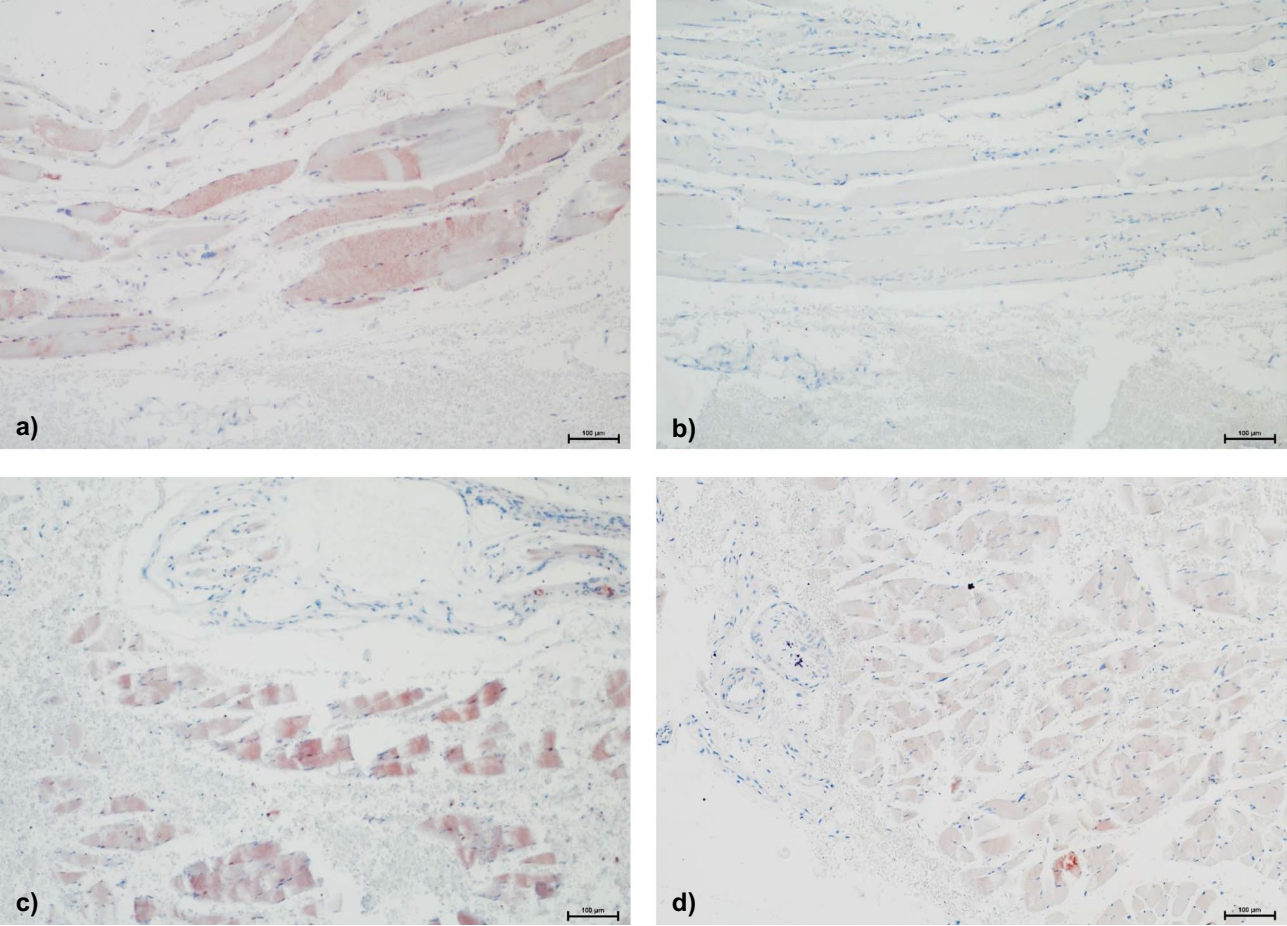
Examples of very inhomogeneous staining results of human skeletal muscle injuries (100-fold magnification). **a** Wound age group D (8–12 h), MMP-9, staining intensity III. **b** Wound age group D (8–12 h), TIMP-1, staining intensity 0. **c** Wound age group B (few min–4 h), MMP-9, staining intensity III. **d** Wound age group B (few min–4 h), TIMP-1, staining intensity III

Table 1 is enclosed for detailed results; examples for staining results are presented in Fig. 2.

- MMP-9Positive staining results for MMP-9 were found in all wound age groups, even in samples with very short survival times. The intensity of staining was mostly equivalent to grade II or even III. However, there was also a considerable number of samples showing no positive staining at all. The share of samples with negative staining was especially large in wound age group D.
- TIMP-1The majority of samples of wound age groups A and B presented positive staining results with an intensity according to grade II or III. In wound age groups C and especially D, the share of samples with negative staining was larger.
- MMP-2Strong positive staining results for MMP-2 according to grades II and III were found in all samples regardless of the wound age group. Negative staining was found in age groups B to D but their share was rather small.

**Table 1:** Staining results of samples of human skeletal muscle injuries. Samples are categorized according to the estimated survival times of the wounds (groups A-D). “*n*” equals the number of samples in each group. The evaluation is based on a staging system used in a previous study [7]: MMP-2: 0 = no visible staining, I = positive staining of extracellular matrix (EM) in the perivascular regions, II = positive staining of EM in larger areas, III = positive staining not only of EM but also of intracellular. MMP-9 and TIMP-1: 0 = no visible staining, I = positive staining of single cells, II = positive staining of cell groups, III = positive staining of large tissue areas

Human—skeletal muscle	A				B				C				D			
	(few min max.)				(few min-4 h)				(4–8 h)				(8–12 h)			
	*n* = 27				*n* = 91				*n* = 11				*n* = 11			
	III	II	I	0	III	II	I	0	III	II	I	0	III	II	I	0
MMP-9	8	8	2	9	18	26	23	24	3	5	2	1	2	1	3	5
TIMP-1	6	12	3	6	19	34	18	20	3	2	3	3	3	1	2	5
MMP-2	4	17	6	0	34	35	18	4	4	3	3	1	5	4	1	1

### Human myocardium

**Fig. 3:**
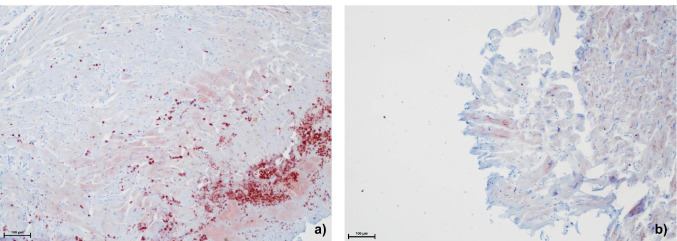
Comparison between staining results (MMP-9, 100-fold magnification) of human myocardium injuries due to **a** infarction with staining intensity III and **b** sharp violence with staining intensity III. In both cases, a survival time according to group A has to be assumed

Table 2 presents the detailed results for human myocardium injuries. Examples for staining results can be found in Fig. 3.

- MMP-9A high number of samples in wound age group A showed staining results with intensities grade II and III. The share of grade III was especially high in wounds that followed an infarction. However, nearly half of the samples in group A showed no positive staining. Similar findings could be observed for group B. The single sample in group D presented no positive staining.
- TIMP-1The majority of samples of wounds in group A showed results with staining intensities grade II and III. Again, the share of grade III was especially high in wounds that followed an infarction. Also, a considerable number of samples showed no positive staining at all. The same accounted for samples in group B. The one sample in group D presented a staining intensity grade I.
- MMP-2Staining results in group A mainly presented intensities grade II and III with a considerable high share of grade III in infarction-derived wounds. In group B, the share of samples with a staining intensity grade I was greater. The sample in group D also presented positive staining with intensity grade I. Negative staining results for MMP-2 were only found in single cases.

**Table 2:** Staining results of samples of human myocardium injuries. Samples are categorized according to the estimated survival times after infliction of wounds (groups A-D). “*n*” equals the number of samples in each group. The evaluation is based on a staging system used in a previous study [6]: MMP-2: 0 = no visible staining, I = positive staining of extracellular matrix (EM) in the perivascular regions, II = positive staining of EM in larger areas, III = positive staining not only of EM but also of intracellular. MMP-9 and TIMP-1: 0 = no visible staining, I = positive staining of single cells, II = positive staining of cell groups, III = positive staining of large tissue areas

Human—myocardium	A				B				C				D			
	(few min max.)				(few min-4 h)				(4–8 h)				(8–12 h)			
	*n* = 52				*n* = 15				*n* = 0				*n* = 1			
	III	II	I	0	III	II	I	0	III	II	I	0	III	II	I	0
MMP-9	11	11	6	24	2	5	1	7	0	0	0	0	0	0	0	1
TIMP-1	18	9	7	18	8	2	1	4	0	0	0	0	0	1	0	0
MMP-2	17	19	13	3	6	4	4	1	0	0	0	0	0	0	1	0

### Rats’ hearts—vital wounds

Table 3 shows the detailed results of rat hearts with vital wounds.

- MMP-9 Only two positive staining results with intensity grade I were observed in one heart with a survival time of 30 min and in one heart with a survival time of 3 h. In all other cases, staining was negative.
- TIMP-1 There was only one heart with a survival time of 1 h that showed discreet positive staining. All other hearts showed no positive staining at all.
- MMP-2 Nine hearts showed positive staining results with intensities grade I and especially grade II. The shortest survival time with positive staining results was 15 min. From the hearts with longer survival times, only one heart with a survival time of 1 h stained positive.

**Table 3:** Staining results of rats’ hearts with vital wounds. Survival time equals the time between injury and fixation of the hearts in formalin. The evaluation is based on a staging system used in a previous study [6]: MMP-2: 0 = no visible staining, I = positive staining of extracellular matrix (EM) in the perivascular regions, II = positive staining of EM in larger areas, III = positive staining not only of EM but also of intracellular. MMP-9 and TIMP-1: 0 = no visible staining, I = positive staining of single cells, II = positive staining of cell groups, III = positive staining of large tissue areas

Rats—vital wounds	Survival time	TIMP-1	MMP-2	MMP-9
H1	5 min	0	0	0
H2	10 min	0	0	0
H3	15 min	0	II	0
H4	30 min	0	II	I
H5	1 h	I	II	0
H6	2 h	0	II	0
H7	3 h	0	II	I
H8	4 h	0	I	0
H9	4 h	0	II	0
H10	1 h	0	0	0
H11	3 h	0	II	0
H12	2 h	0	I	0
H13	5 min	0	0	0
H14	10 min	0	0	0
H15	15 min	0	0	0
H16	30 min	0	0	0

### Rats’ hearts—postmortem-inflicted wounds

**Fig. 4:**
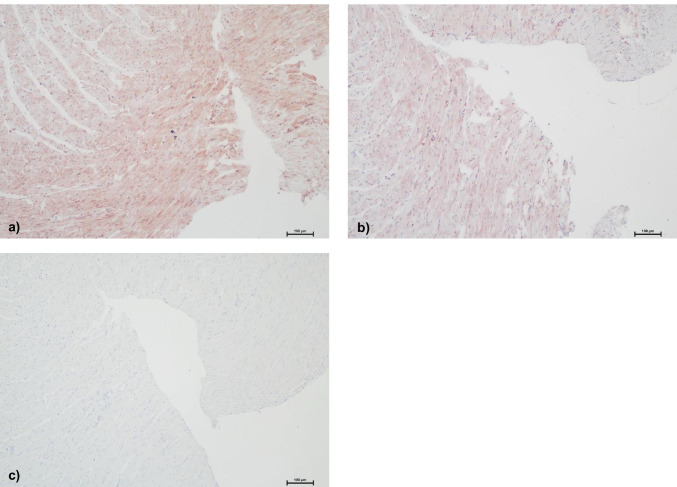
Example of staining results of rats’ hearts with postmortem-inflicted injuries (100-fold magnification). **a** MMP-2. **b** MMP-9. **c** TIMP-1. The depicted heart (CH2) was injured 3 h after excision and fixed in formalin after another 3 h. MMP-2 and MMP-9 each present intense staining, whereas TIMP-1 shows no positive staining at all

Table 4 shows the detailed results of rats’ hearts with postmortem-inflicted wounds, and examples of staining results are presented in Fig. 4.

- MMP-9 Three hearts presented positive staining results with intensities grade I and II. The time spans between the end of heartbeat and the infliction of the wounds varied between 0 min and 3 h In all cases, and the time span after wound infliction was 3 h.
- MMP-2 Three hearts presented negative staining results, including the two hearts with wounds inflicted 20 min after the heart had stopped beating. All the other hearts showed positive staining with intensities grade I and especially grade II.
- TIMP-1 None of the hearts presented positive staining results.

**Table 4:** Staining results of rats’ hearts with postmortem-inflicted wounds. The time spans equal the time between death and injury, and between injury and fixation of hearts in formalin. The evaluation is based on a staging system used in a previous study [6]: MMP-2: 0 = no visible staining, I = positive staining of extracellular matrix (EM) in the perivascular regions, II = positive staining of EM in larger areas, III = positive staining not only of EM but also of intracellular. MMP-9 and TIMP-1: 0 = no visible staining, I = positive staining of single cells, II = positive staining of cell groups, III = positive staining of large tissue areas

Rats—post mortem wounds	Time intervals after death and after injury	TIMP-1	MMP-2	MMP-9
CH1	10 min—stab—3 h	0	II	I
CH2	3 h—stab—3 h	0	II	II
CH3	3 h—stab—3 h	0	I	0
CH4	stab—3 h	0	II	I
CH5	10 min—stab—3 h	0	0	0
CH6	stab—3 h	0	I	0
CH7	20 min—stab—4 h	0	0	0
CH8	20 min—stab—4 h	0	0	0

## Discussion

We addressed the challenges that go along with forensic wound age estimation as illustrated above by a unique sample collection comprised in the study: Not only were we able to include two different types of tissues (skeletal muscle and myocardium) from two species (humans, rats), the sample set also includes wounds with a defined wound age and reliably postmortem-inflicted wounds thanks to the Langendorff system. Thus, we not only took advantage of controlled experimental conditions in the rat model but also examined the applicability of the results gained on human tissue samples.

Despite this broad approach, the presented results are very inhomogeneous and show great “scattering”. We found positive staining for all the tested markers in a considerable number of human samples regardless of their origin and the wound age. However, the same accounts for negative staining results (see Fig. 2 for examples of human skeletal muscle). If any, there was a slight tendency of more intense staining results towards cases with a “younger” wound age: Apparently, the number of samples showing no positive staining or less intense staining increased with higher wound age, implying that the markers we evaluated occur shortly after the infliction of a wound and disappear rather fast. Similar findings have also been published by Wang et al. [22]: In skin wounds of mice, high levels of MMP-9/MMP-2 seem to suggest an earlier stage of wound healing. The authors came to the conclusion that an increased expression and activation of MMP-2 might be important for the inflammation phase following an injury rather than being crucial for the process of wound healing.

Regarding the results of human samples with wound age group A (very short survival time, few min max.), the share of those with staining intensities grade III was slightly higher in heart muscle samples than in skeletal muscle samples. This might lead to the assumption that wound healing and the emergence of the evaluated markers kick in faster in myocardium. However, a closer look on the myocardium samples revealed that the high staining intensities are mainly found in infarction-derived injuries. Though the differences were rather discreet, it is still obvious that staining of MMP-2, MMP-9, and TIMP-1 in younger wound age groups was more intense in cases with an underlying infarction compared to those with injuries of other origins. Therefore, we assume that in infarctions, the already existing inflammation might have caused an earlier expression and/or activation of the markers, which goes along with other research findings [23]. Since particularly inner organs, but also skin in certain cases, might present acute or chronic illnesses, statements on wound age based on the detection of an inflammation-related marker have to be made cautiously. Referring to the review by Li et al. [2], it appears that “[…] wound age estimation is an intricate and multifactorial problem […]” which means that numerous intrinsic and extrinsic factors must be taken into account when trying to determine the age and vitality of a wound.

Compared to human samples, rat hearts with vital wounds presented rather few positive staining results especially for markers MMP-9 and TIMP-1. In addition, positive staining was found earliest in cases with a wound age of 15 min. In many of the human cases, a shorter survival time has to be assumed. At least in part this alleged contradiction might be connected to the fact that tissues/cells can “survive” the death of the individual for some time. In case of the rats’ hearts, “wound age” refers to the time between infliction of wounds and fixation of the hearts in formalin. Under such circumstances, the death of all cells occurs almost simultaneously and at the same point of time as the death of the individual, i.e. the heart. Since such a sudden stop of all intracellular activities does not apply for the human cases, wound age processes that have been triggered at or shortly before the time of death might still have proceeded and caused the emergence of the investigated markers some time later—simulating a faster occurrence in human tissue. In addition, the lack of blood in the Langendorff system might result in a delayed activation and/or expression of MMPs and TIMPs since relevant mediators might be missing.

Dunjic et al. [5] already stated that after the individual death of a person, some cells are still active. Referring to this review, the time span during which the cells are active also seems to depend upon the type of tissue. According to Tsujimoto et al. [24], the time of cell death is also influenced by ATP levels. Fibroblasts in human skin samples could be analyzed several days postmortem [5]. In this context, supravital reactions even several hours after the individual death can be explained. White blood cells seem to remain active for up to 12 h postmortem, which questions the presence of an inflammation as a vital reaction after injury. Additionally, Alaeddini et al. [4] and Jennings et al. [25] also described different intervals of survival due to different tissue mechanisms and stated that necrosis starts in defined regions of every organ, such as the subendocardial regions in the human heart. Compared to other organs, skeletal muscle tissue seems to show postmortem ultrastructural changes quite late [26]. In a study on lamb muscle, Sylvestre et al. [27] were able to provide evidence of postmortem activity of MMP-2. High levels of pro-MMP-2 and of active MMP-2, but also of active MMP-2, were detected not only on the day of slaughter, but also 21 days later in samples that had been stored at 4 °C. The high levels of MMP-2 led to the assumption that MMP-2 is involved in the degradation of the tissue. The problem of distinguishing between vital wounds and postmortem-inflicted wounds was also described in a review by Cecchi et al. in 2010 [28] who pointing out that a variability in their detection makes many markers unreliable when it comes to this question. Furthermore, the methods used for detecting a marker, e.g. polymerase chain reaction or IHC, seem to have an impact upon the results.

Against this background, the behavior of the markers tested in our study is not surprising. The occurrence of MMP-2 and MMP-9 in vital and postmortem-inflicted wounds does not show obvious differences. Merely for TIMP-1, some interesting results were obtained: Although there were many positive staining results in human samples, rats’ hearts with vital wounds presented positive staining for TIMP-1 only in one case with a wound age of 1 h. Furthermore, there were no positive staining results for TIMP-1 in rats’ hearts with postmortem-inflicted wounds. The overall picture of these findings suggests that TIMP-1 is not as sensitive as MMP-2 and MMP-9, implying a possible use as a vitality marker. To verify this hypothesis, TIMP-1 needs to be tested on human muscle samples with reliably postmortem-inflicted wounds. Unfortunately, such samples are difficult to obtain and were therefore not comprised in the study at hand.

## Limitations

Our study is subjected to some limitations: Our collected samples of human tissue only include wounds with a wound age up to 12 h. The maximum post-infliction time span of rats’ hearts accounted for 240 min. We therefore have no information about the behavior of the markers when used on wounds that are days or even weeks old. In addition, the available data for the tissue samples drawn during autopsies underlie some uncertainties, especially with a view to the exact time of the infliction of the wounds. Uninjured control samples were already included in the preceding study [6], whereas samples of human skeletal muscle with postmortem-inflicted wounds and a reasonable postmortem interval are difficult to obtain and therefore could not be examined. Furthermore, the number of rat tissue samples examined in this study might seem comparably low. We resigned from increasing the number due to ethical aspects.

## Conclusion

Our study again demonstrates the challenges that go along with forensic wound age estimation and the establishment of new markers. Despite our complex approach of examining MMP-9, MMP-2, and TIMP-1 on both human and rat muscle tissue, as well as on vital and postmortem-inflicted wounds, we were faced with disappointing results. Though unexpected findings in a preceding study on rat hearts were quite promising, the results of the far more comprehensive sample collection show a very inhomogeneous picture leading to the conclusion that the markers do not meet the complex requirements of forensic wound age and wound vitality estimation. Only TIMP-1 might be of use when trying to differentiate between vital and postmortem-inflicted wounds but it needs to be tested on postmortem-inflicted wounds of human muscle samples.

Overall, it became clear again that a profound understanding of the usefulness of potential markers can only be achieved by examining a variety of samples. The sample collection needs to include vital wounds and postmortem-inflicted wounds. When working with an animal model, human control samples are indispensable; otherwise, the transferability of results remains questionable. The same accounts for different types of tissues. Finally, potential influences of acute or chronic illnesses have to be kept in mind when interpreting analytical results.

## Data Availability

Data sharing is not applicable to this article as no datasets were generated or analyzed during the current study.
